# End-of-Life Materials Used as Supplementary Cementitious Materials in the Concrete Industry

**DOI:** 10.3390/ma13081954

**Published:** 2020-04-22

**Authors:** Adrian Ionut Nicoara, Alexandra Elena Stoica, Mirijam Vrabec, Nastja Šmuc Rogan, Saso Sturm, Cleva Ow-Yang, Mehmet Ali Gulgun, Zeynep Basaran Bundur, Ion Ciuca, Bogdan Stefan Vasile

**Affiliations:** 1 Department of Science and Engineering of Oxide Materials and Nanomaterials, Faculty of Applied Chemistry and Materials Science, University Politehnica of Bucharest, 011061 Bucharest, Romania; adrian.nicoara@upb.ro (A.I.N.); elena_oprea_93@yahoo.co.uk (A.E.S.); 2 National Research Center for Micro and Nanomaterials, Faculty of Applied Chemistry and Materials Science, University Politehnica of Bucharest, 060042 Bucharest, Romania; 3 Department of Geology, Faculty of Natural Sciences and Engineering, University of Ljubljana, Aškerčeva 12, 1000 Ljubljana, Slovenia; mirijam.vrabec@geo.ntf.uni-lj.si (M.V.); nastja.rogan@guest.arnes.si (N.Š.R.); 4 Department for Nanostructured Materials, Jozef Stefan Institute, Jamova cesta 39, SI-1000 Ljubljana, Slovenia; saso.sturm@ijs.si; 5 Materials Science and Nano-Engineering Program, Sabanci University, Orta Mahalle, Üniversite Caddesi No:27, 34956 Tuzla–İstanbul, Turkey; cleva.ow-yang@sabanciuniv.edu (C.O.-Y.); m-gulgun@sabanciuniv.edu (M.A.G.); 6 Nanotechnology Application Center (SUNUM), Sabanci University, Orta Mahalle, Üniversite Caddesi No:27, 34956 Tuzla–İstanbul, Turkey; 7 Department of Civil Engineering, Ozyegin University, Nişantepe District, Orman Street, Çekmeköy, 34794 Istanbul, Turkey; zeynep.basaran@ozyegin.edu.tr; 8 Faculty Materials Science and Engineering, University Politehnica of Bucharest, 060042 Bucharest, Romania; ion.ciuca11@gmail.com

**Keywords:** construction debris, cement, recycling, circular economy, eco-friendly concretes, fly ash (FA), silica fume (SF), palm oil fuel ash (POFA), rice husk ash (RHA), sewage sludge ash (SSA) and sugarcane bagasse ash (SBA), mine tailings, marble dust, construction and demolition debris (CDD)

## Abstract

A sustainable solution for the global construction industry can be partial substitution of Ordinary Portland Cement (OPC) by use of supplementary cementitious materials (SCMs) sourced from industrial end-of-life (EOL) products that contain calcareous, siliceous and aluminous materials. Candidate EOL materials include fly ash (FA), silica fume (SF), natural pozzolanic materials like sugarcane bagasse ash (SBA), palm oil fuel ash (POFA), rice husk ash (RHA), mine tailings, marble dust, construction and demolition debris (CDD). Studies have revealed these materials to be cementitious and/or pozzolanic in nature. Their use as SCMs would decrease the amount of cement used in the production of concrete, decreasing carbon emissions associated with cement production. In addition to cement substitution, EOL products as SCMs have also served as coarse and also fine aggregates in the production of eco-friendly concretes.

## 1. Introduction

In the face of rapidly expanding urbanization, environmental sustainability represents a serious challenge for the construction industry, whose consumption of concrete requires a significant quantity of natural reserves worldwide and necessitates the development of alternative materials and sources. The fabrication of concrete consumes around 27 billion tonnes of feedstock, representing 4 tonnes of concrete for each person every year. By 2050, concrete production will be four times higher than in 1990. Aggregates and binder (i.e., cement) represent around 60%–80% and 10%–15% of the total weight of concrete, respectively [1]. Along with processing of substantial amount of aggregates and around 2.8 billion tonnes of cement products per year, concrete generates approximately 5%–7% of the global total carbon dioxide emissions. By 2025, around 3.5 billion tonnes of carbon dioxide is foreseen to be released to the atmosphere during cement production. One solution for more sustainable production can be harvesting locally available end-of-life (EOL) and/or recyclable materials [1,2].

**Figure 1:**
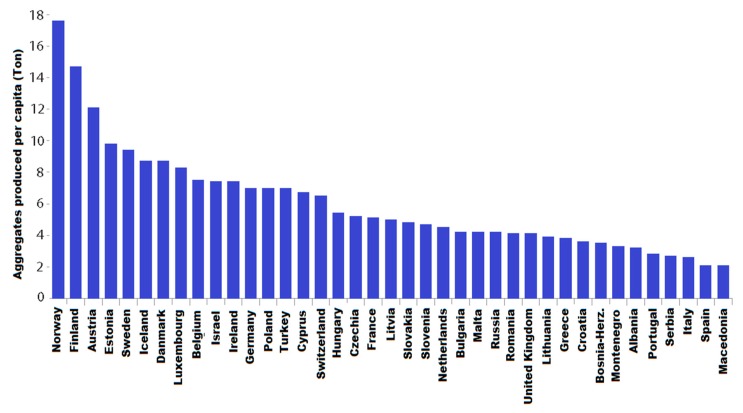
Aggregates production, in tonnes per capita, in 39 countries [3].

Global quarry practices to obtain coarse aggregates have substantially modified the ecological equilibrium. Figure 1 shows the amount, in tonnes of aggregates produced per capita in 39 countries. For the sustainable future of our planet, it is essential to find substitutes for virgin materials to harvest for producing binder and aggregates necessitated by the construction industry. Meanwhile, a lot of EOL materials are disposed of in open fields as landfill. One example of this kind of waste is construction and demolition debris with enormous potential for recycling as a profitable recycled concrete aggregate (RA) [2,3].

RA can be found in almost all developed and developing countries as a result of the demolition of older buildings and structures. Moreover, in war-afflicted regions in other parts of the world, a large number of buildings have been destroyed by bomb attacks. Such buildings have become impractical and are of no-value, but offer significant potential as material sources for reconstruction projects. Currently, a significant volume of RA is being disposed of as zero-value debris. Therefore, RA can play an important role in sustainable development. RA has recently become an important domain in construction as substitutes for natural aggregate raw materials. Several studies [2,4,5,6,7,8,9] claimed that the incorporation of RA in concrete production would alter the hardened properties. Several studies demonstrated that 100% substitution of aggregates with RA in concrete is unacceptable, due to a significant decrease in the hardened strength varying from 15 to 25% [2].

**Figure 2:**
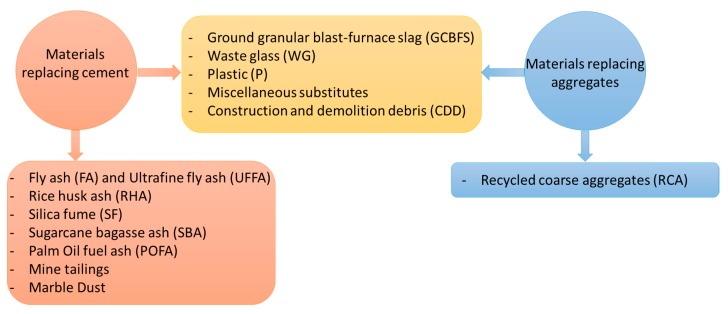
Most common industrial by-products used as substitutes [26].

In addition, concrete has been produced with traditional supplementary cementitious materials (SCMs) that possess a high pozzolanic activity [10], such as fly ash (FA) [11,12,13,14], silica fume (SF) [15,16,17] and ground granulated blast slag (GGBS) [2,18,19,20,21] that yielded notable improvement in strength and durability. Numerous industrial solid by-products containing calcareous siliceous, and aluminous materials (fly ash, ultrafine fly ash, silica fume, etc.), along with some natural pozzolanic materials [22] (volcanic tuffs, diatomaceous earth, sugarcane bagasse ash, palm oil fuel ash, rice husk ash, mine tailings, etc.) can be used as SCMs, because they possess cementitious and/or pozzolanic properties [23]. The abundance of these classes of materials and their broad diversity in chemical and physical composition compel the development of a common strategy for their application in concrete production industry (see Figure 2) [24,25].

Although traditional SCMs have been attractive due to their superior long-term durability [27], sustainable SCMs can also be developed to decrease the quantity of cement required for concrete production with lower ecological impact. One example is the incorporation of industrial EOL by-products into conventional cement. Concrete containing up to 30% of cement substituted by SCMs has been regarded as environmentally-friendly concrete [2,10,28]. In recent years, the use of SCMs and/or natural pozzolans has increased in the concrete industry, due to their superior long-term performance. Consequently, there is a strong interest in activating large amounts EOL materials to replace traditional SCMs as different sustainable resources that are otherwise deemed as of-zero-value [2,10].

## 2. Supplementary Cementitious Materials (SCMs)

SCMs play a pivotal role in concrete performance across many civilizations. They encompass a wide spectrum of alumino-silicious materials, including natural or processed pozzolans and industrial by-products, like GGBS, FA/UFFA and SF [29]. In spite of broad variations in properties across the various types of SCMs, they share in common the capacity to react chemically in concrete and form cementitious binders replacing those obtained by OPC hydration [30]. The key feature of SCMs is their pozzolanicity, i.e., their capability to react with calcium hydroxide (portlandite, CH) aqueous solutions to form calcium silicate hydrate (C–S–H) [31].

In the right proportion, SCMs can improve the fresh and hardened properties of concrete, especially the long-term durability. The use of SCMs in concrete composition is an ancient technique [32,33,34], as evidenced by the widespread use of natural pozzolans, like volcanic ash, in Greek and Roman civilizations. The testament to their efficacy is the persistence of an important number of constructions built using pozzolanic materials that are still standing today [30]. 

The most frequently employed SCMs in the cement industry are discussed below: fly ash (FA) and/or ultrafine fly ash (UFFA) [35], silica fume (SF) [36,37] and natural pozzolanic materials like rice husk ash (RHA) [38,39,40], sugarcane bagasse ash (SBA) [41,42,43], sewage sludge ash (SSA) [44,45], palm oil fuel ash (POFA) [46,47,48,49], mine tailings [50,51,52,53], marble dust [54,55,56,57,58], construction and demolition debris (CDD) [59,60,61].

### 2.1. Silica Fume (SF)

Silica fume (SF) used to be EOL products harvested from industrial processes. However, lately the demand for the silica fume for high-performance concrete has increased so strongly that there are now foundries dedicated to producing SF for ultra -high-performance concrete. SF may be in the form micro-silica, condensed silica fume or volatilized silica [26]. It is a fine powder produced in silicon foundries, where it is ultimately condensed from the vapour phase upon cooling [10]. As a consequence, SF is composed almost entirely of very small round particles of amorphous SiO_2_, whose fineness contributes to a relatively high pozzolanic activity [62]. The size distribution of regular SF particles is on the order of 0.1 µm, where the majority of the particles—more than 95%—should be smaller than 1 µm. They are about 100 times smaller than cement particles, with a specific surface of SF is around 20,000 m^2^/kg, therefore their specific surface is 10 to 20 times larger than that of other pozzolanic materials. Considering its characteristics, SF is a very reactive pozzolanic material [63]. The use of SF can notably increase mechanical properties of concrete due to effective filling and pozzolanic properties [64].

SF’s most important effect on concrete is on the short- and long-term strength and long-term durability [26]. SF is claimed to be capable of increasing the bonding between cement paste and aggregates at the interfacial transition zone (ITZ). With a small particle size, SF not only improves the packing in the ITZ but also uses the localized CH in the ITZ during pozzolanic reaction, in combination with additional C–S–H. The net effect is creating better adhesion between the cement paste and aggregate. The high surface area of SF also provides a large reaction surface for enhancing the hydration process, resulting in improved density [26]. SF’s influence on the improved density, or reduced macro porosity, also stems from its fluidized bed effect. The perfectly round fine particles of SF facilitate denser packing in concrete. Improved flow properties of concrete in the fresh state reduce bleeding and segregation, leading to superior performance of fresh and hardened concrete. Additional pozzolanic activity helps fill the excess water volume during further hydration [26,65].

**Figure 3:**
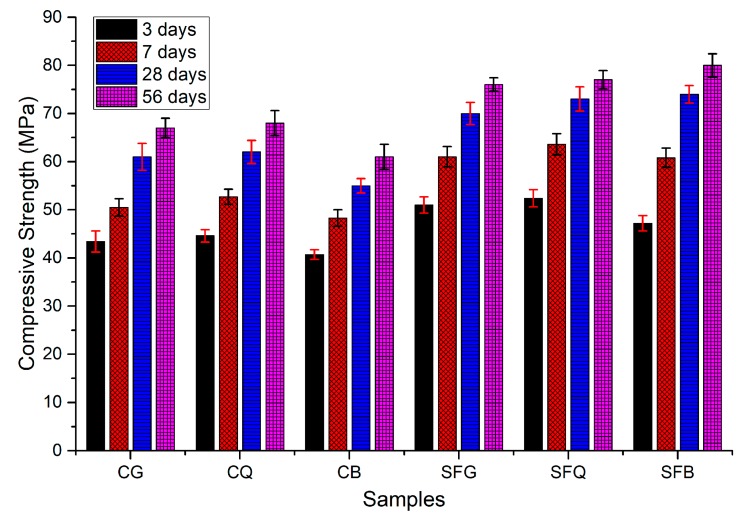
Compressive strength test results (the specimens made with only cement as binder were noted with the acronyms CG, CQ and CB for concrete with granite, with quartz and with basalt aggregates, respectively). Those containing silica fume were called SFG, SFQ and SFB, accordingly [66].

A study by Seong Soo Kim et al. [66] demonstrated the improvement in compressive strength due to SF incorporation. Three types of coarse aggregates (basalt, quartzite and granite) were used to produce different concrete samples. The main binder used was OPC (according to ASTM C150), while SF was used to substitute 10 wt. % of cement. Figure 3, from this study, demonstrates the increase in compressive strength increased for all coarse aggregates types (i.e., basalt, quartzite and granite), when SF was added (see Figure 3).

**Figure 4:**
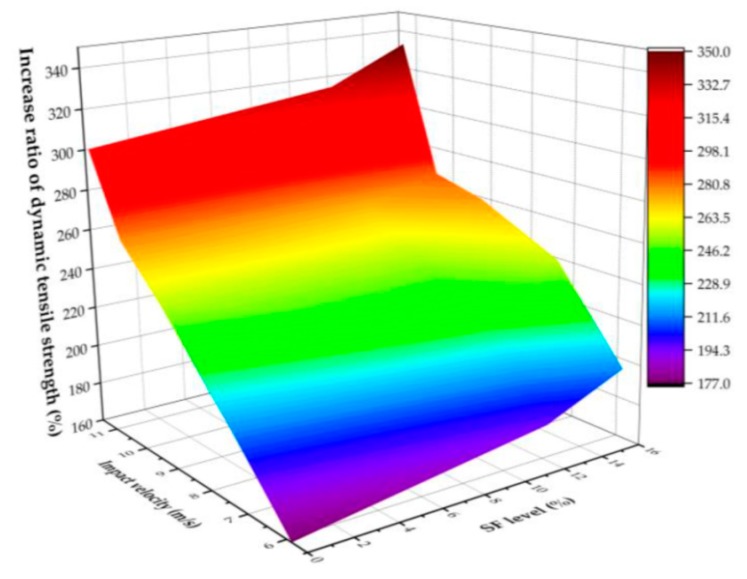
Dynamic tensile strength under different impact velocities and silica fume levels [67].

Tensile performance was investigated in another study, in which 3 different series of concrete samples (see Table 1) with 0 wt.%, 12 wt.% and 16 wt.% SF as a cement substitute were produced and tested [67]. The results indicated that SF added to concrete yielded superior mechanical properties under dynamic tensile loading. Moreover, the dynamic tensile strength of samples rose with the silica fume (SF) amount, as well as with strain rate. In addition, the strain rate sensitivity of concrete augmented with SF appeared to have increased in comparison to concrete without SF (see Figure 4) [67].

SF additions to concrete improve several other physical and chemical properties besides the mechanical strength: lower adverse environmental impact, decreased permeability, increased corrosion protection for steel bars and improved resistance against the sulphate and chemicals attacks [68,69].

### 2.2. Fly Ash (FA)

Fly ash (FA) is a by-product generated from coal-fired power plants. It is a fine, volatile powder emitted from chimneys alongside flue gases [10]. There are three distinct types of FA, including Class N, Class F and Class C, produced by burning black coal or brown coal, respectively. Class C and Class F are used in production of building materials, like lightweight aggregate, concrete, bricks, etc. (see Table 2, Table 3 and Table 4) [26,70]. There have been numerous studies, in which fly ash to be used as SCM in concrete have been analysed and classified according to types and characteristics. The American Society for Testing and Materials (ASTM) produced the initial report on standards for applying FA more than 40 years ago, ASTM C 618 [26].

Coal-fired power plants produce significant solid wastes that contained FA. About 700 million tons of FA every year throughout the world can be used in cement and/or concrete production, thanks to its pozzolanic activity. Replacing with FA in cement or concrete results in gross energy requirement (GER), carbon dioxide emissions (Global Warming Potential: GWP) and natural resource consumption [71,72]. Despite these advantages, the development of optimal composition of concrete with a high volume fraction of fly ash (HVFA) remains a challenge, due to the broad variation in chemical composition (lime, sulphates, alkalis and organics), fineness and mineralogy of fly-ash [71].

**Figure 5:**
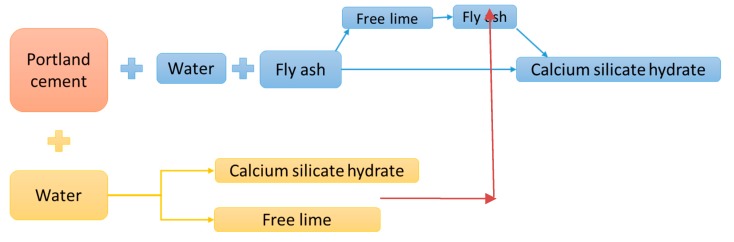
Reaction of fly ash (FA) in cement [70].

In recent years, the integration of fly ash as a partial substitute of cement in concrete is a common procedure. The amount of FA to substitute the cement for regular applications is restricted to 15%–35% (depending on the type of FA) by mass of the total amount of cementitious material. However, even at these levels, the use of FA has overcome some critical issues focused on sustainable construction. FA’s pozzolanic activity is related to the presence of amorphous SiO_2_ and Al_2_O_3_ in its composition [73]. An active FA reacts with Ca(OH)_2_ during the cement hydration process and forms supplementary C–S–H and calcium aluminate hydrate (C–A–H) [73,74]. These hydration products allow formation of a denser matrix, resulting in better strength and superior durability (see Figure 5) [73,74].

**Figure 6:**
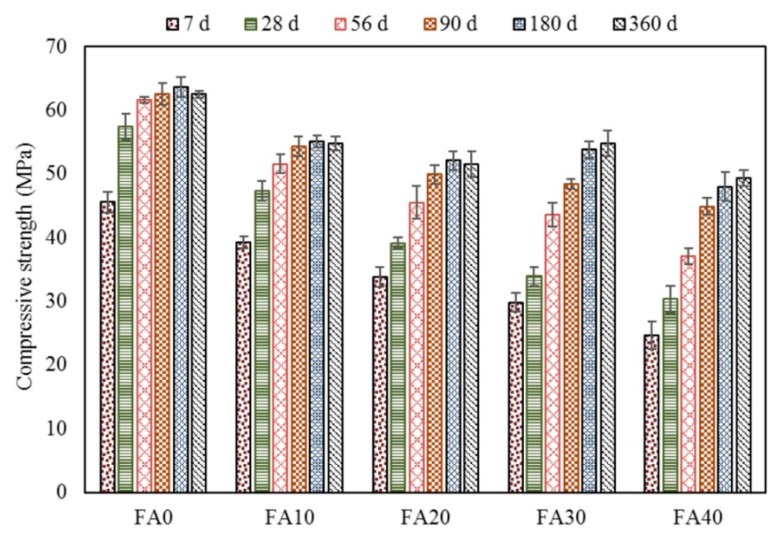
Compressive strength development [75].

Ashish Kumer Saha [75] studied the use of Class F fly ash as a partial substitute of binder in concrete. A set of five samples was cast—a control sample without FA and 4 samples consist of 10, 20, 30 and 40% of FA as a substitute for cement. The water-to-cement ratio and the volume of superplasticizer were maintained constant for all 5 samples. The compressive strength (see Figure 6) of the FA mixtures showed lower early compressive strength than the ones of the control samples. The positive influence of FA additions could be observed at the longer hydration times, i.e., longer than 28 days of hydration. The small sized FA particles with high surface area and high percentage of amorphous silica phase provided the pozzolanic activity, leading to enhanced strength for longer periods of hydration. In addition, the spherical shape of FA particles improves the fresh state properties by increasing the workability of the concrete mix. 

**Figure 7:**
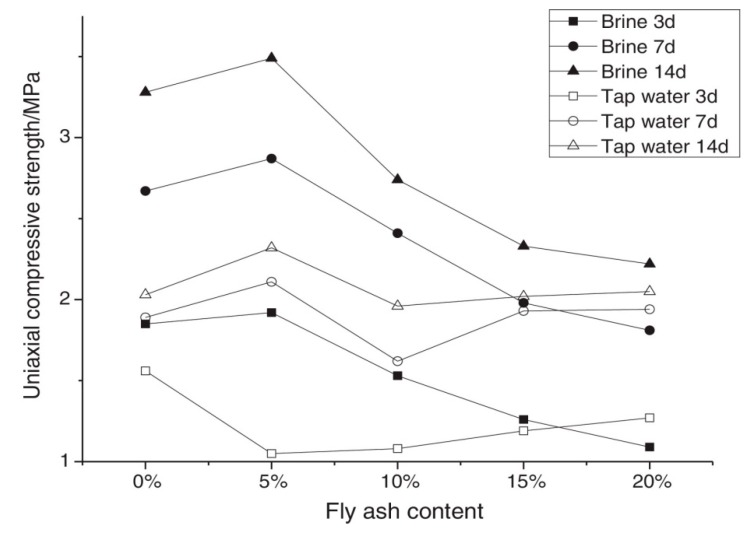
The compressive strength of the specimen and the quantity FA content [76].

Bingqian Yan et al. [76] studied the changes of mechanical properties of cement mortar with an admixture of FA and lime. As brine water is used in the preparation of filling slurry of Sanshandao Gold Mine, the chloride ions in the slurry have a great negative effect on the strength of the backfill. Figure 7 shows that the uniaxial compressive strength of the cement mortar sample with an amount of FA of 5% is 0.2 MPa better than the one without FA. When the amount continued to increase, the uniaxial compressive strength of cement mortar declined with increasing FA content. Analysis revealed that the activity of FA under the excitation of lime and brine water was restricted and large amounts of FA use presented a negative effect on the strength development and on cementing properties of binder [76].

FA is in the form of spherical particles composed of many phases—amorphous and also crystalline compounds, mostly silicon, calcium, aluminium and iron oxides. The versatility of fly ash for production of different types of cements is attributed to its physical features, chemical properties and phase composition [77,78,79].

### 2.3. Other Pozzolanic Ashes

Rice Husk Ash (RHA). An agricultural by-product that is suitable for cement replacement in rice-growing regions is Rice Husk Ash (RHA) [38]. The cementitious characteristics of RHA unappreciated before the 1970s [26]. RHA is the combustion residue from rice husks, which are the stiff outer layer that accumulates during de-husking of paddy rice. Every tonne of paddy rice can yield around 200 kg of husk, which produces about 40 kg of ash after combustion. It is known that rice plants consume H_4_SiO_4_ from underground water that exists in saturated zones beneath the earth surface. H_4_SiO_4_ at this point is polymerized and leads to development of amorphous silica in the husks [80]. During combustion of the organic compounds, CO_2_ is produced along with the silica remaining in the ash leftovers. The researchers have demonstrated that the principal chemical composition of rice husk ash consists of biomass-driven silicon dioxide (SiO_2_). Table 5 summarizes its typical chemical composition and some of its properties. As a result that the nature of silica in rice husk ash is sensitive to processing conditions (see Table 5), the ash obtained through open-field burning or uncontrolled combustion in furnaces generally includes a high percentage of crystalline silica minerals, like tridymite or cristobalite, with inferior reactivity. The highest amount of amorphous silica is obtained when RHA is burnt at temperatures ranging from 500 to 700 °C [81]. The superior reactivity of RHA is due to its large amount of amorphous silica, which has high surface area due to the porous architecture of the host material. RHA can be used as a substitute in Portland cement (acceptable up to 15%), thanks to its pozzolanic activity. Fine RHA can increase the compressive strength of cement paste and can lead to preparation of mortars with low porosity [26,39]. 

As a cement substitute, the application of RHA in concrete production has advantages and disadvantages [39]. Improved compressive strength of concrete is one of the essential advantages of using RHA as substitute. Recent studies have highlighted important benefits of replacing cement with RHA in small percentages [26]. In the context of durability, the use of RHA as a substitute in concrete production can lead to notable improved water permeability resistance, Cl^−^ penetration and sulphate deterioration [83].

**Figure 8:**
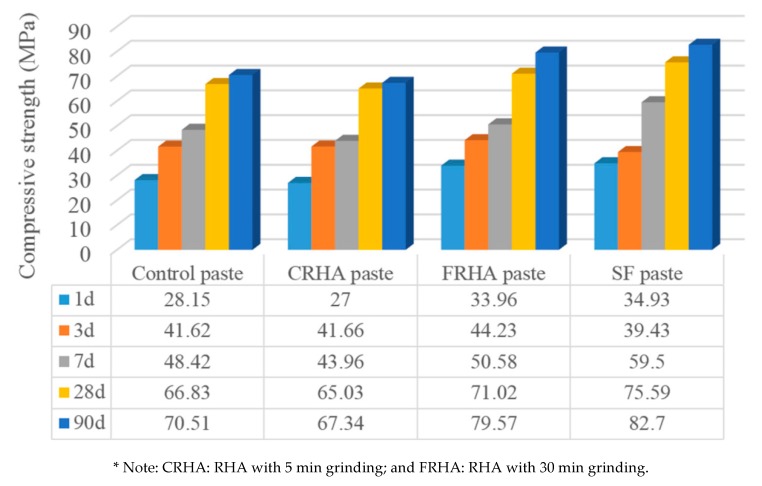
Compressive strength of studied pastes [83].

Weiting Xu et al. [83] compared the pozzolanic impact of SF and ground RHA as SCMs on the properties of composite cement pastes and concretes. The authors evaluated mechanical property, workability, durability and microstructure. In the composite cement pastes, the binder (OPC) was substituted with SF and finely ground RHA (named FRHA) at 5%, 10%, 15%, 20%, 25% and 30%, respectively by mass. They reported that the optimal substitution percentage of SF and RHA were 10% by weight of cement in pastes and concretes. Compressive strength for this composition was evaluated (see Figure 8) [83].

**Figure 9:**
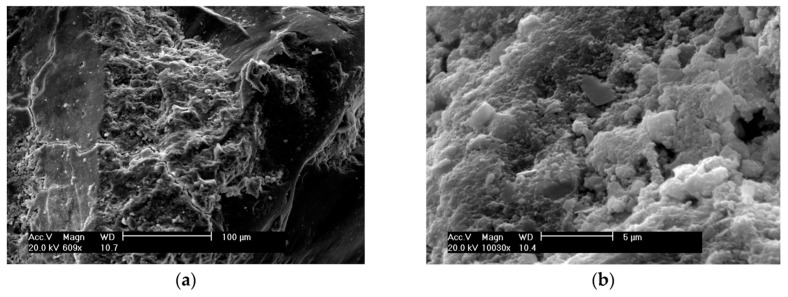
SEM images of concrete containing SF [83]. (**a**) Global Image—100 µm (**b**) Detailed image 5 µm.

**Figure 10:**
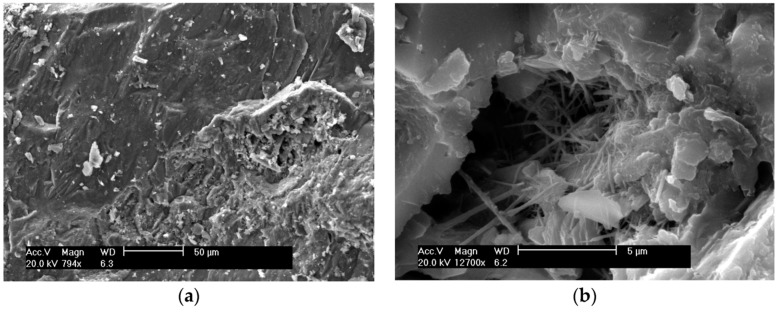
SEM images of concrete containing SF [83]. (**a**) Global Image—100 µm (**b**) Detailed image 5 µm. In view of the empirical results [83], it appeared that FRHA exhibits similar pozzolanic and rheological activity to SF and can lead to notable improvement in the properties of a cementitious system.

It can be observed that the sample with coarse rice husk ash (CRHA) had the lowest compressive strength at all curing ages, which could be because of the bigger particle size and small surface area of coarse RHA particles. Tests results showed that addition of FRHA to the paste led to an increased compressive strength compared to the control concrete, as a result of the growing specific surface area and pozzolanic activity of RHA. In addition, the morphological dissimilarity may also be implicated by the differences in compressive strength between the concrete specimens (see Figure 9 and Figure 10) [83].

Sugarcane bagasse ash (SBA) is a by-product of producing juice from sugar cane by crushing the stalks of the plants (see Table 6). The addition of SBA in concrete production can decrease the hydration temperature up to 33%, when 30% of OPC is substituted by SBA [42]. Furthermore, water permeability considerably decreases when compared to control concrete samples. With the aim of superior compressive strength, OPC was substituted in the range from 15% to 30%. It was also claimed that SBA aids the reduction of ASR expansion in concrete, by binding alkalis [26]. Table 6 lists the properties of SBA.

**Figure 11:**
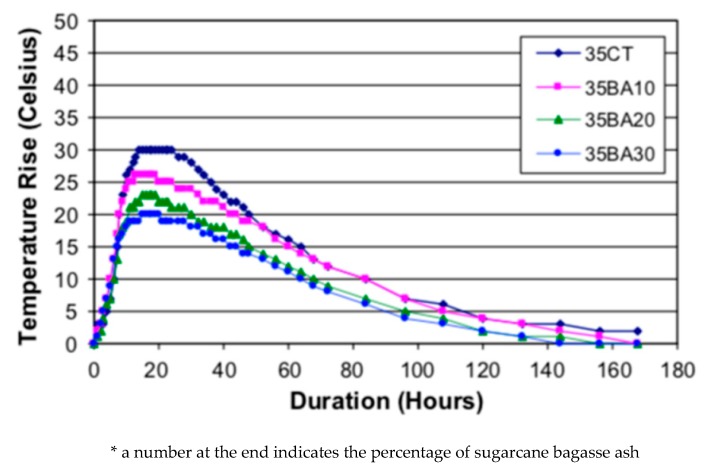
Semi-adiabatic temperature rise (°C) in concrete containing SBA as substitute [82].

Use of SBA as limited cement substitute reduces hydration heat, compared to that of the control reference. The decrease of semi-adiabatic temperature rise (°C) is proportional to the percentage of SBA replaced (see Figure 11). Therefore, SBA may be used to control the temperature in mass concrete pouring [82].

**Figure 12:**
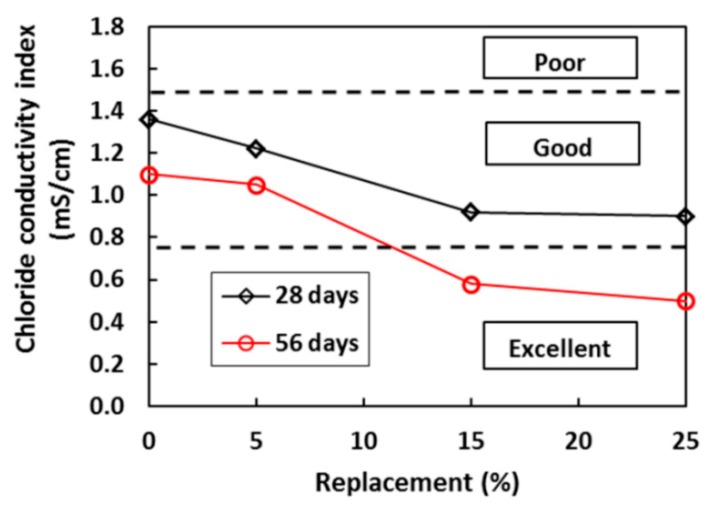
Effect of SBA on the chloride conductivity index of concrete [82].

SBA incorporation improved concrete durability. A composite concrete with additions of different amounts of SBA was studied in the context of chloride attack (see Figure 12), as well as gas and water permeability [41,82].

Wastes of different sources have been investigated for their possibility in re-use, to reduce their environmental impact, in landfill volume and decomposition by-products [84]. Sewage sludge ash (SSA) is an urban waste that may be used as fertilizer, as well as a cement substitute [26,85,86,87]. SSA was not only considered as SCM in blended cements but also in a large scale of building materials like pave-stones, tiles, bricks, light aggregates production. The impact of SSA in mortar was a decrease in the compressive strength, when SSA was applied as partial cement substitute. Therefore, use of SSA as an SCM was shown to be limited, in the construction domain. The cement community does not include SSA in the group of pozzolanic materials [88].

Palm oil fuel ash (POFA) is an important cash-crop in tropical countries, especially in Malaysia and Indonesia [82]. For every 100 t of fresh fruit bunches handled, there will be about 20 t of nut shells, 7 t of fibres and 25 t of empty bunches released from the mills. POFA can be used in concrete either as aggregates, SCM or as filler material [89,90]. Comparable to RHA and SBA, the amorphous SiO_2_ (around 76%) content of POFA offers relatively high pozzolanic activity, when used as binder in concrete production. Even though a few performance parameters of concrete (especially setting time and strength) are negatively influenced by POFA, several studies claimed that palm oil fuel ash may be appropriate in different applications [91,92]. It may be an important resource in developing countries, although more studies are certainly needed, to support the use of POFA in structural applications [82,93,94]. 

Mine tailings. The amount of mine tailings has grown excessively with an ever increasing demand for metal and mineral resources [95]. Mining wastes are produced during mineral extraction by the mining industry and is at present one of the largest waste flows worldwide [25,95,96]. Mine tailings are finely ground in the time of mineral processing and separation of minerals of interest [96]. A significant part of milling processes and separation procedure uses water as the transport medium. Therefore, tailings obtained by mining enterprises usually consist of small particle slurries with high water content, flow ability and poor mechanical stability [95]. Mine tailings are deposited in dammed ponds along with industrial wastewater or as thickened pastes in piles close to the extraction sites [96]. At present, their principal use is as backfill in mined-out underground areas or as deposits in tailing ponds. As such, they pose potential long-term risks as environmental pollution. However, use of tailings is not only relevant to environmental conservation, but can also benefit the mining industry. These solid wastes contain compounds with potential pozzolanic properties and can decrease the amount of cement used to produce concrete, reducing simultaneously the ecological impact of the cement and mining industries. An additional benefit of mine tailings is that they are already finely ground. Most of the other SCMs require mechanical grinding, as a pre-treatment for use, to improve their reactivity. However, mine tailings already come with favourable physical and chemical properties as particle dimensions, crystal structures and even surface properties [96].

Therefore, depositories of tailings have lately gained global attention [95]. However, despite the potential of mine tailings as substitutes in the production of cementitious materials, there is a striking lack of studies regarding this topic in the cement and concrete literature.

Marble dust. Marble is a finely crystallized metamorphic rock originating from the low-intensity metamorphism of calcareous and dolomitic rocks. Calcium carbonate (CaCO_3_) can form up to 99% of the total amount of this carbonated rock. Additional phases may also include SiO_2_, MgO, Fe2O_3_, Al_2_O_3_ and Na_2_O and, in minor ratio, MnO, K_2_O, P_2_O_5_, F, Cu, S, Pb and Zn [97]. For a long time, marble has been a significant building material. For instance, annual production of marble in Turkey accounts for 7 Mt and represents 40% of the global storage; Turkey has an annual total production of block marble of ca. 1,500,000 m^3^, generating approximately 375,000 m^3^ of marble powder [98]. Throughout the shaping, sawing and polishing operations, around 20%–25% of processed marble is converted into powder or lumps. As a result, dumps of marble dust have become an important environmental issue worldwide [99]. Marble powder (MP) has successfully been demonstrated as a viable SCM in self-compacting concrete (SCC). The research proved that marble powder used as mineral substitute of cement can enhance some properties of fresh concrete and/or hardened concrete [100]. In the cement-related literature, there are just a few research studies related to the application of marble powder in concrete or mortar production. Thus, more detailed studies are needed in order to define the properties of concretes or mortars with marble powder. The use of marble powder in ternary cementitious blends demands further caution to remove or reduce its adverse effects on the fresh properties of self-compacting concrete and/or mortar [98].

Construction and demolition debris (CDD) constitute one of the massive flows of solid waste generated from municipal and commercial activities of modern society [101]. Usually, CDD are in the shape of brick bats, mortars, aggregates, concrete, glass, ceramic tiles, metals and even plastics. They must be mechanically sorted according to size and quality level. They are then crushed down to desired size [102]. It is essential to study the life cycle of construction materials to develop a global understanding of sustainable building construction and the feasible use of CDD as SCMs for OPC substitution. The life cycle of some construction materials, such as concrete, has been analysed to evaluate their environmental consequences. While substantial effort has been applied to LCA-based sustainability assessment of construction materials and buildings, the specialty literature needs more detailed studies regarding the LCA of recycled construction materials, ones that take into account both process and supply chain-related outcomes as a whole [103]. The sheer mass and heterogeneity of CDD materials and absence of data classification across non-standardized tracking systems have led to new challenges [101]. In addition, the lack of knowledge about the possible savings and implications correlated with recycling of this kind of construction materials from the life cycle outlook still limits their use [103].

## 3. Life Cycle Assessment (LCA)

**Figure 13:**
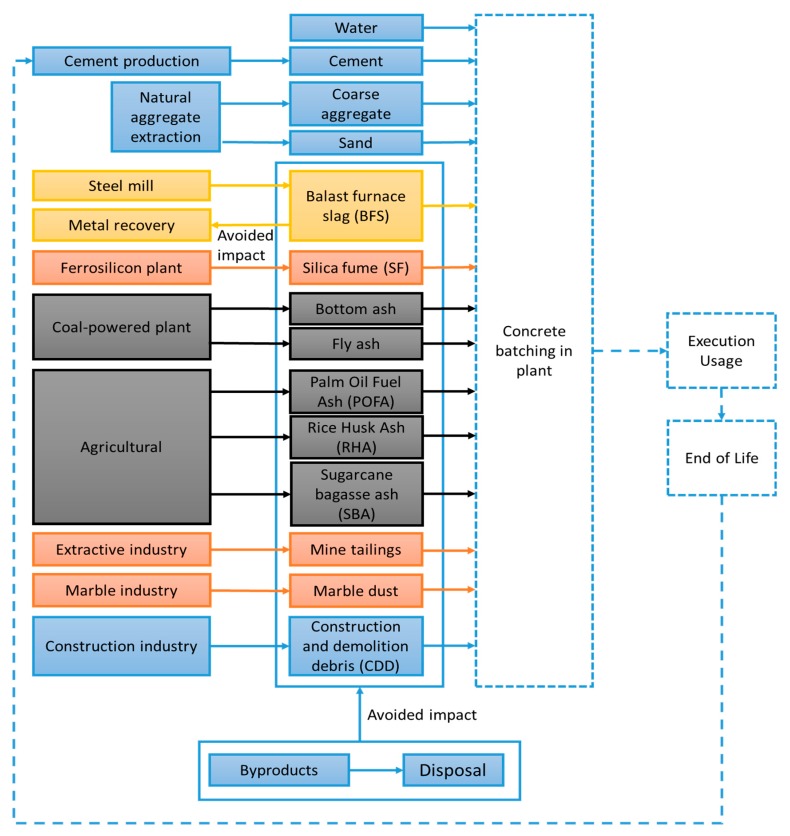
Cradle-to-gate life cycle assessment (LCA): studied system boundaries of the concretes (plain line—included processes; dashed line—non-included processes) [111].

A life-cycle assessment (LCA) is a method for assessing all the potential environmental impacts of a product, process, or activity over its entire life cycle [104]. Several LCA studies have focused on the sustainability of concrete [105,106]. It is important to integrate recycled EOL products at the beginning of concrete’s life cycle, and re-valorise it at the end of concrete’s life cycle in another production process or even maybe, for the concrete production industry (see Figure 13) [107,108,109,110].

Sustainability through re-use of accessible economic and social resources is a way to attain equilibrium with the environment, while ensuring long-term development and endurance [26]. A beneficial difference for the environment could result from substituting cement and sand with by-products or EOL products from intersectoral industrial activities. By this way, we may be able to reduce the adverse environmental impacts stemming from cement (74%–93%) and sand (0.3%–2%) consumption in the total LCA of EOL material-based concretes. The minimum contribution of sand to the entire environmental assessment of concrete makes this issue important to concrete design [111,112,113].

With regard to the LCA of concrete, 4 aspects should be considered: (1) design, (2) production/execution, (3) usage and (4) end-of-life disposal. Structural concrete has life expectancy differentiated by application, such as in pillars, beams and walls. While the durability over time for foundation or load-bearing structural elements is 50–300 years, the corresponding lifetime for cover walls is only 20–50 years [111]. Therefore, sufficient data is not presently available for the EOL stage of structural concrete and its disposal conditions [111,114,115,116].

The necessity for further LCA studies on the treatment and re-use of construction waste is clear. Instead of being released into the environment, it can be re-valorised in the life cycle of new designs of concrete. Use of waste like fly ash, blast furnace slag and other mineral admixtures as a binder for the production of concrete is becoming common in the construction industry. Replacing the principal factor responsible for the negative environmental effects of concrete is the key to generating an ecologically beneficial life cycle for concrete [107,117,118].

With regards to economic impact, regenerating alternative EOL materials for binder components in concrete would decrease the cost of construction without sacrificing performance. Other costs can also be considered, such as those concerning the source and transport of the alternative SCM materials, controlled combustion process and also savings as a result of diversion, such as disposal management. Consequently, the environmental advantages will reduce the enormous demand for Portland cement per unit volume of concrete, in addition to a concurrent and meaningful reduction in Greenhouse Gas (GHG) emissions [108,119,120].

## 4. Conclusions

Concrete represents one of the most widely used construction materials worldwide by volume. Portland cement production is highly energy intensive, and emits significant amounts of CO_2_ through the calcination process, which contributes substantial adverse impact on global warming. Efforts are needed to design and develop more ecologically friendly concrete with improved performance in strength and durability.

SCMs are frequently applied in concrete mixtures as a substitute for clinker in cement or cement in concrete. This approach yields concrete with reduced cost, decreased environmental impact, higher long-term strength and better long-term durability. Presently the two most common SCMs are silica fume and fly ash. As a by-product of the silicon and ferrosilicon alloy fabrication, SF contains more than 90% SiO_2_ and is present as spheres with average diameters about 100 times smaller than cement particles. The large specific surface of SF, ca. 20,000 m^2^/kg, is 10 to 20 times greater than that of other pozzolanic materials, imbuing it with high pozzolanic activity and fluidization properties.

FA has also been widely used in concrete mix design, due to its growing availability and substantial environmental complications appeared by release of fly ash. Physical characteristics of FA can differ on the nature of coal, rank, mineral matter chemistry and mineralogy, furnace design, furnace operation and method of particulate control, while chemical characteristics are relatively insensitive those factors. FA has been used in the manufacturing of bricks, concrete and cement-composites like columns, beams, slabs, columns, sheets, pipes, wall panels, etc. Typically, about 25% of FA is used as a substitute for cement to achieve effective final products. FA increases durability, workability, density and workability of concrete, while simultaneously decreasing water demand, porosity and permeability of concrete.

In the present, the use of industrial EOL materials in concrete has been demonstrated. However, it is clear that more research is needed to assess the feasibility of long-term performance, develop more ecologically sound production, in addition to quality assessment of these materials. When ashes of high quality can be regularly obtained with reduced financial and most importantly environmental costs, their use in the engineering domain will become more widespread. Technical and economic performances of alternative SCM is evident, proving that along with the studying of the material’s mechanical and physico-chemical properties, the review of its life cycle is as well important and will mention whether it will be environmentally feasible to apply the SCM admixture at the total scale of the life cycle of concrete.

## Figures and Tables

**Table 1:** The proportion of concrete constituents with different SF levels by weight [67].

Mass of Concrete Constituents (kg/m^3^)					
Series	Water	Cement	SF	Fine Aggregate	Aggregate
I	210.00	389.00	–	614	1141
II	210.00	340.80	48.20	614	1141
III	210.00	326.28	62.72	614	1141

**Table 2:** Type of fly ash as per American Society for Testing and Materials [26,70].

Type	CaO Amount, [%]	Properties
class C	≥10	cementitious, pozzolanic, hydraulic
class F	≤10	Pozzolanic
class N	is not commonly used in construction because of the existence of clay and shale	

**Table 3:** Type of fly ash as per S 3812-1981.

Type	SiO_2_+Al_2_O_3_+Fe_2_O_3_ Fraction, [%]
Grade I	≥70
Grade II	≥50

**Table 4:** Type of fly ash based on boiler operations.

Type	Short Name	Forming Temperature
Low temperature fly ash	LT	≤900 °C
High temperature fly ash	HT	≤1000 °C

**Table 5:** Typical chemical and physical properties of Rice Husk Ash (RHA) [82].

Chemical Composition *, [%]					
SiO_2_	Al_2_O_3_	Fe_2_O_3_	CaO	MgO	K_2_O
93.4	0.05	0.06	0.31	0.35	1.4
**Physical Properties**					
Fineness—median particle size (µm)			8.6		
Specific gravity			2.05		
Pozzolanic activity index (%)			99		
Water absorption (%)			104		

* Minor Constituents Not Given.

**Table 6:** Usual chemical and physical properties of sugarcane bagasse ash (SBA) [82].

Chemical Composition *, [%]					
SiO_2_	Al_2_O_3_	Fe_2_O_3_	CaO	MgO	K_2_O
65.3	6.9	3.7	4.0	1.1	2.0
**Physical Properties**					
Fineness—median particle size (µm)			5.1		
Specific gravity			1.8		
Blaine fineness (m^2^/kg)			900		

* Minor constituents not given.
